# Supplementary material for: Differential Coding of Fruit, Leaf, and Microbial Odours in the Brains of Drosophila suzukii and Drosophila melanogaster
Source: Insects. 2025 Jan 15;16(1):84. doi: 10.3390/insects16010084 (PMC11766258; doi:10.3390/insects16010084)
Supplement: Supplementary file 1 [file insects-16-00084-s001.zip › insects-3356548-supplementary.pdf]

Supplementary Materials

Differential coding of fruit, leaf and microbial odours in the brains of *Drosophila suzukii* and *Drosophila melanogaster*.

Claire Dumenil[1,2], Gülsüm Yildirim[1], Albrecht Haase[1,3,*]

[1] Centre for Mind/Brain Sciences (CIMeC), University of Trento, Rovereto, Italy;
[2] Faculty of Agricultural, Environmental and Food Sciences, Free University of Bozen-Bolzano, Bolzano, Italy; clairefrancenicolle.dumenil@unibz.it
[3] Department of Physics, University of Trento, Trento, Italy;

* Correspondence: albrecht.haase@unitn.it

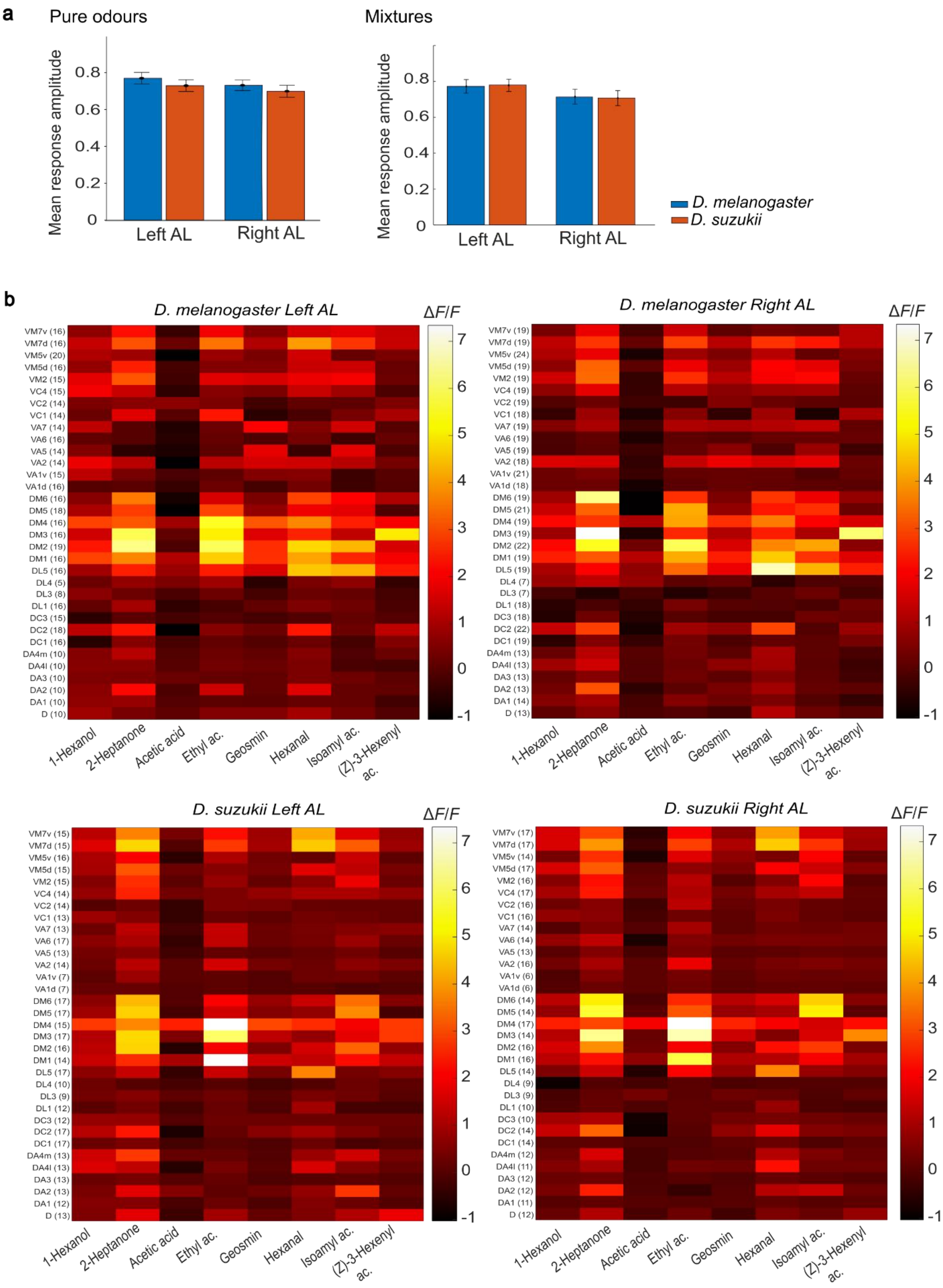

a
Pure odours
Mixtures
Mean response amplitude
0
0.2
0.4
0.6
0.8
Left AL
Right AL
D. melanogaster
D. suzukii
b
D. melanogaster Left AL
D. melanogaster Right AL
D. suzukii Left AL
D. suzukii Right AL
ΔF/F
-1
0
1
2
3
4
5
6
7
VM7v (16)
VM7d (16)
VM5v (20)
VM5d (16)
VM2 (15)
VC4 (15)
VC2 (14)
VC1 (14)
VA7 (14)
VA6 (16)
VA5 (14)
VA2 (14)
VA1v (15)
VA1d (16)
DM6 (16)
DM5 (18)
DM4 (16)
DM3 (16)
DM2 (19)
DM1 (16)
DL5 (16)
DL4 (5)
DL3 (8)
DL1 (16)
DC3 (15)
DC2 (18)
DC1 (16)
DA4m (10)
DA4l (10)
DA3 (10)
DA2 (10)
DA1 (10)
D (10)
VM7v (19)
VM7d (19)
VM5v (24)
VM5d (19)
VM2 (19)
VC4 (19)
VC2 (19)
VC1 (18)
VA7 (19)
VA6 (19)
VA5 (19)
VA2 (18)
VA1v (21)
VA1d (18)
DM6 (19)
DM5 (21)
DM4 (19)
DM3 (19)
DM2 (22)
DM1 (19)
DL5 (19)
DL4 (7)
DL3 (7)
DL1 (18)
DC3 (18)
DC2 (22)
DC1 (19)
DA4m (13)
DA4l (13)
DA3 (13)
DA2 (13)
DA1 (14)
D (13)
VM7v (15)
VM7d (15)
VM5v (16)
VM5d (15)
VM2 (15)
VC4 (14)
VC2 (14)
VC1 (13)
VA7 (13)
VA6 (17)
VA5 (13)
VA2 (14)
VA1v (7)
VA1d (7)
DM6 (17)
DM5 (17)
DM4 (15)
DM3 (17)
DM2 (16)
DM1 (14)
DL5 (17)
DL4 (10)
DL3 (9)
DL1 (12)
DC3 (12)
DC2 (17)
DC1 (17)
DA4m (13)
DA4l (13)
DA3 (13)
DA2 (13)
DA1 (12)
D (13)
VM7v (17)
VM7d (17)
VM5v (14)
VM5d (17)
VM2 (16)
VC4 (17)
VC2 (16)
VC1 (16)
VA7 (14)
VA6 (14)
VA5 (13)
VA2 (16)
VA1v (6)
VA1d (6)
DM6 (14)
DM5 (14)
DM4 (17)
DM3 (14)
DM2 (16)
DM1 (16)
DL5 (14)
DL4 (9)
DL3 (9)
DL1 (10)
DC3 (10)
DC2 (14)
DC1 (14)
DA4m (12)
DA4l (11)
DA3 (12)
DA2 (12)
DA1 (11)
D (12)
1-Hexanol
2-Heptanone
Acetic acid
Ethyl ac.
Geosmin
Hexanal
Isoamyl ac.
(Z)-3-Hexenyl ac.

Figure S1. Symmetry assessment of odour response patterns in *D. suzukii* and *D. melanogaster*.

Mean ± SEM response amplitude measured in the right and left antennal lobes (AL) averaged over the eight single odours (a) and the four single odours and their mixtures (b) in *D. suzukii* (orange) and *D. melanogaster* (blue). c) Mean ± SEM response amplitude during a 3 s stimulus with eight single odours in each glomerulus on the left and right AL in *D. melanogaster* (upper heatmaps) and *D. suzukii* (lower heatmaps). The number of replicates is indicated between brackets next to each glomerulus (y-axis). The colour gradient indicates inhibitions (black, $\Delta F/F < 0$) up to the largest activations (white, $\Delta F/F > 7$). ac. = acetate.

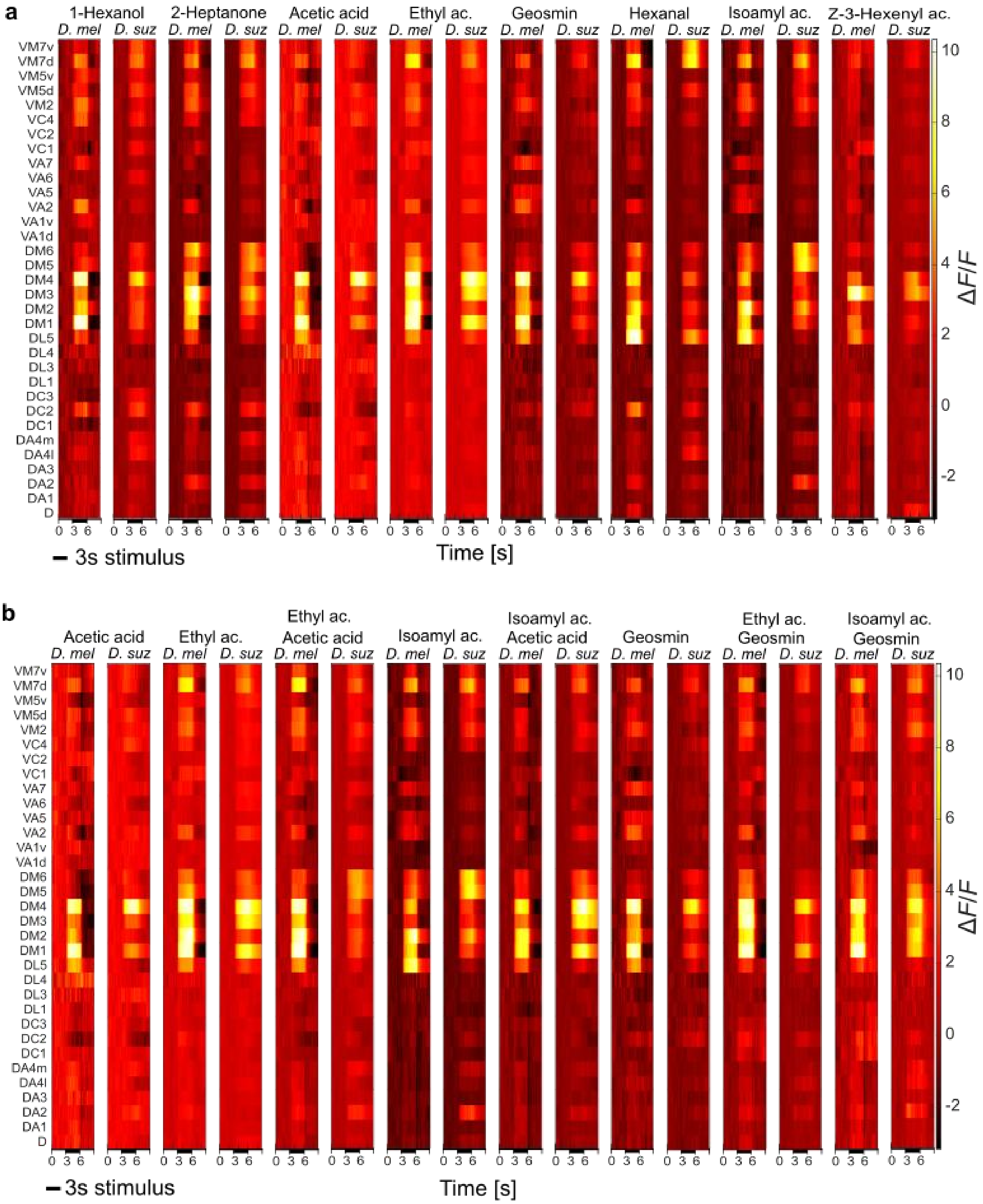
a
1-Hexanol
2-Heptanone
Acetic acid
Ethyl ac.
Geosmin
Hexanal
Isoamyl ac.
Z-3-Hexenyl ac.
D. mel
D. suz
VM7v
VM7d
VM5v
VM5d
VM2
VC4
VC2
VC1
VA7
VA6
VA5
VA2
VA1v
VA1d
DM6
DM5
DM4
DM3
DM2
DM1
DL5
DL4
DL3
DL1
DC3
DC2
DC1
DA4m
DA4l
DA3
DA2
DA1
D
0 3 6
3s stimulus
Time [s]
ΔF/F
b
Acetic acid
Ethyl ac.
Ethyl ac. Acetic acid
Isoamyl ac.
Isoamyl ac. Acetic acid
Geosmin
Ethyl ac. Geosmin
Isoamyl ac. Geosmin

Figure S2. Odour response maps for eight odorants and their mixtures in *D. suzukii* and *D. melanogaster.*

Subject-averaged temporal response maps to eight odour compounds before, during, and after a 3 s stimulus plotted for individual glomeruli for eight odours (a) and 4 odours and their mixtures (b). $\Delta F/F$ was normalised, the same scale was applied to all heatmaps to allow for comparison between species and odours. 'ac.' acetate.

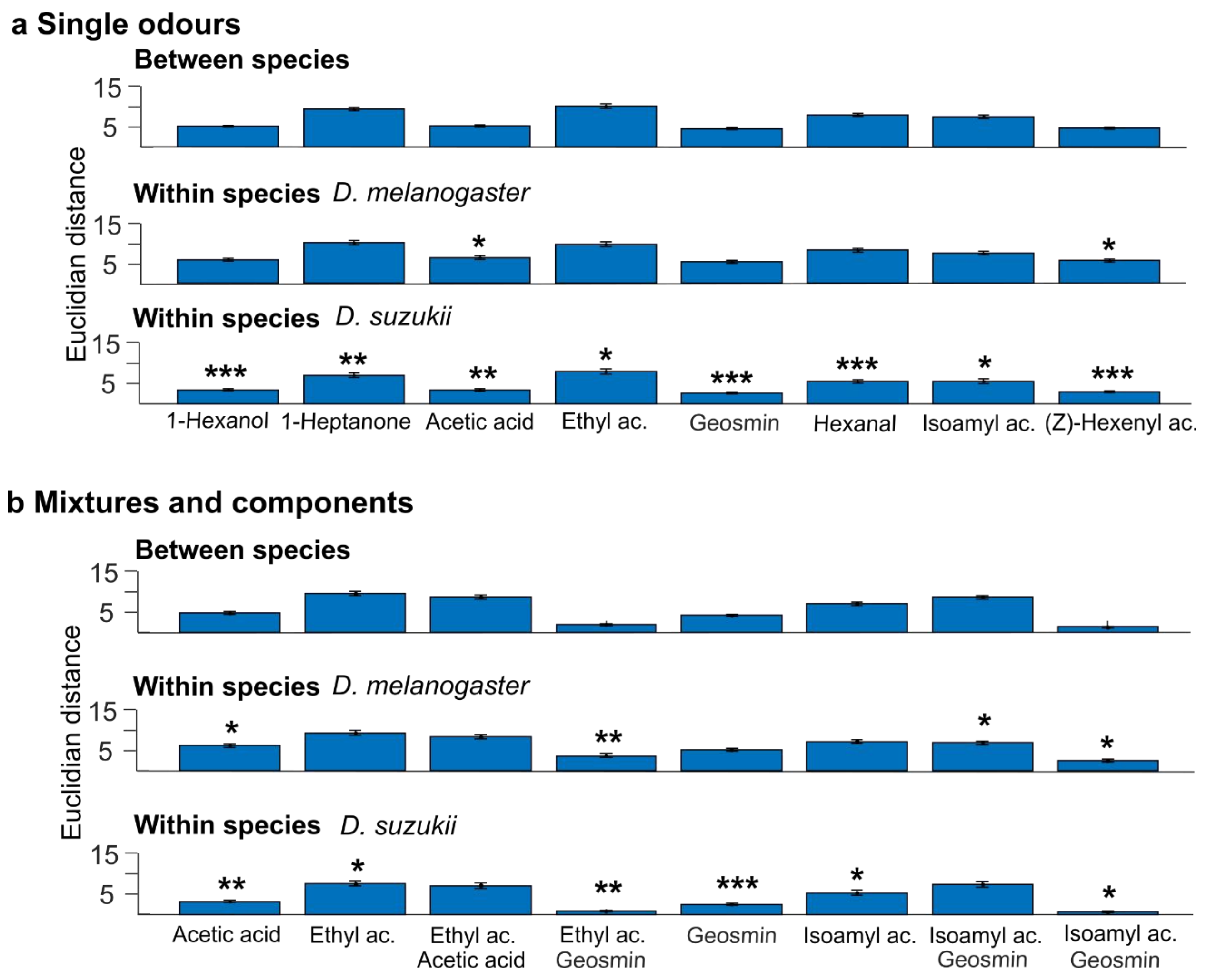

Figure S3. Euclidean distance analysis for odour response difference between species vs. within species.

Mean ± SEM of the Euclidean distance (ED) of odour-response amplitudes averaged over all fly pair combinations: between species, within *D. melanogaster*, and within *D. suzukii* in (a) the pure odour experiments and (b) for the mixtures and their components. Significant statistical differences (multiple comparison analysis with FDR correction) in the comparison of between-species ED and within-species ED are labelled according to their significance probabilities as * $p < 0.05$, ** $p < 0.01$, ***, $p < 0.001$.

Table S1. 2-way ANOVA of the normalised glomerular volume

	Sum Sq.	*df*	Mean Sq.	*F*	*p*
**Species**	3.3E-07	1	3.3E-07	0.0017	0.97
**Glomeruli**	0.25	31	0.008	42	1E-126
**Interaction**	0.034	31	0.0011	5.7	5.3E-19
**Error**	0.11	575	1.9E-4		
**Total**	0.39	638			

Table S2. Multiple comparison analysis of the normalised glomerular volumes

Glomerulus	-95% CI	Mean	+95% CI	*P*	P_{FDR}
**D**	-0.028	-0.016	-0.0038	0.01	0.046
**DA1**	-0.014	-0.0015	0.011	0.81	0.9
**DA2**	-0.009	0.0032	0.015	0.61	0.9
**DA3**	-0.018	-0.0058	0.0064	0.35	0.88
**DA4L**	-0.011	0.0015	0.014	0.81	0.9
**DA4M**	-0.01	0.002	0.014	0.74	0.9
**DC1**	-0.019	-0.0065	0.0056	0.29	0.86
**DC2**	-0.051	-0.039	-0.027	5.2E-10	8.4E-09
**DL1**	-0.014	-0.0021	0.01	0.73	0.9
**DL3**	-0.0078	0.0043	0.016	0.48	0.88
**DL4**	0.0093	0.021	0.034	5.5E-4	0.0035
**DL5**	-0.013	-0.00083	0.011	0.89	0.91
**DM1**	-0.016	-0.0038	0.0083	0.53	0.88
**DM2**	0.029	0.041	0.053	4.9E-11	1.6E-09
**DM3**	0.02	0.032	0.044	3.8E-07	4.0E-6
**DM4**	-0.016	-0.0037	0.0084	0.55	0.88
**DM5**	-0.027	-0.015	-0.0024	0.019	0.076
**DM6**	-0.011	0.0012	0.013	0.84	0.9

**VA1D**	-0.0023	0.0099	0.022	0.11	0.35
**VA1V**	-0.014	-0.0014	0.011	0.83	0.9
**VA2**	-0.016	-0.004	0.0082	0.52	0.88
**VA5**	-0.023	-0.011	0.0013	0.08	0.28
**VA6**	-0.0094	0.0027	0.015	0.66	0.9
**VA7**	-0.0069	0.0053	0.017	0.39	0.88
**VC1**	-0.017	-0.0046	0.0075	0.46	0.88
**VC2**	-0.015	-0.0025	0.0096	0.68	0.9
**VC3**	-0.011	0.0007	0.013	0.91	0.91
**VM2**	-0.014	-0.0015	0.011	0.81	0.9
**VM5D**	-0.04	-0.028	-0.016	8.9E-06	7.1E-5
**VM5V**	-0.0067	0.0054	0.018	0.38	0.88
**VM7D**	0.006	0.018	0.03	0.0035	0.019
**VM7V**	-0.016	-0.0042	0.0079	0.5	0.88

Table S3. 4-way ANOVA of the mean response amplitudes to pure odours

**Source**	**Sum Sq.**	**df**	**Mean Sq.**	**F**	**p**
**Species**	0.0012	1	0.0012	2.8E-4	0.99
**Side**	12	1	12	2.8	0.094
**Odour**	1800	7	260	60	6.5E-86
**Glomerulus**	9400	32	290	67	0
**Species:side**	0.13	1	0.13	0.03	0.86
**Species:odour**	330	7	47	11	1.3E-13
**Species:glomerulus**	860	32	27	6.1	2.3E-25
**Side:odour**	9.6	7	1.4	0.31	0.95
**Side:glomerulus**	230	32	7.1	1.6	0.014
**Odour:glomerulus**	3300	224	15	3.3	2.4E-55
**Error**	57000	13155	4.4		
**Total**	75000	13499			

Table S4. Multiple comparison analysis of the species dependence for individual glomerular response averaged over the pure odours

Glomerulus	-95% CI	Mean	+95% CI	*P*	P_{FDR}
**D**	-0.56	-0.18	0.2	0.35	0.56
**DA1**	-0.29	0.093	0.48	0.63	0.83
**DA2**	-0.46	-0.086	0.29	0.66	0.83
**DA3**	-0.26	0.12	0.5	0.53	0.76
**DA4l**	-0.6	-0.21	0.17	0.27	0.49
**DA4m**	-0.62	-0.24	0.14	0.21	0.46
**DC1**	-0.29	0.034	0.36	0.84	0.92
**DC2**	-0.15	0.17	0.48	0.3	0.49
**DC3**	-0.7	-0.33	0.027	0.07	0.26
**DL1**	-0.38	-0.022	0.34	0.9	0.96
**DL3**	-0.65	-0.19	0.26	0.41	0.61
**DL4**	-0.17	0.31	0.8	0.2	0.46
**DL5**	0.9	1.2	1.5	1.3E-13	2.1E-12
**DM1**	0.24	0.57	0.89	6.6E-4	0.0055
**DM2**	0.88	1.2	1.5	5.2E-14	1.7E-12
**DM3**	-0.092	0.23	0.56	0.16	0.46
**DM4**	-0.75	-0.43	-0.11	0.0083	0.046
**DM5**	-0.55	-0.24	0.079	0.14	0.46
**DM6**	-0.53	-0.21	0.11	0.21	0.46
**VA1d**	-0.48	-0.05	0.38	0.82	0.92
**VA1v**	-0.42	0.005	0.43	0.98	0.98
**VA2**	0.027	0.36	0.69	0.034	0.14
**VA5**	-0.33	0.013	0.36	0.94	0.97
**VA6**	-0.69	-0.37	-0.044	0.026	0.12

**VA7**	-0.14	0.2	0.54	0.25	0.49
**VC1**	-0.26	0.074	0.41	0.67	0.83
**VC2**	-0.4	-0.07	0.26	0.68	0.83
**VC4**	-0.5	-0.17	0.15	0.29	0.49
**VM2**	0.18	0.51	0.83	0.0023	0.015
**VM5d**	-0.35	-0.034	0.29	0.84	0.92
**VM5v**	-0.48	-0.17	0.14	0.28	0.49
**VM7d**	-0.54	-0.22	0.096	0.17	0.46
**VM7v**	-0.98	-0.66	-0.34	5.4E-5	5.9E-4

Table S5. Multiple comparison analysis of the species dependence for individual pure odour responses averaged over all glomeruli

**Odour**	**-95% CI**	**Mean**	**+95% CI**	***P***	**P_{FDR}**
**1-Hexanol**	-0.15	0.025	0.19	0.78	0.78
**2-Heptanone**	-0.27	-0.099	0.072	0.26	0.41
**Acetic acid**	-0.33	-0.16	0.015	0.074	0.2
**Ethyl acetate**	-0.21	-0.04	0.13	0.64	0.74
**Geosmin**	0.022	0.19	0.36	0.027	0.11
**Hexanal**	0.16	0.33	0.5	1.4E-4	0.0011
**Isoamyl acetate**	-0.25	-0.084	0.087	0.34	0.45
**(Z)-3-Hexenyl acetate**	-0.049	0.12	0.29	0.16	0.33

Table S6. Multiple comparison analysis of Euclidean distances between species vs. within species for pure odours

	Between species	*D. melanogaster*			*D. suzukii*		
	Mean ± SEM	Mean ± SEM	*P*	P_{FDR}	Mean ± SEM	*P*	P_{FDR}
**1Hexanol**	4.8 ± 0.22	5.5 ± 0.31	0.054	0.072	3.2 ± 0.25	6.10E-06	3.20E-05
**2-Heptanone**	8.9 ± 0.42	9.5 ± 0.54	0.34	0.42	6.7 ± 0.57	0.0024	0.0064
**Acetic acid**	4.9 ± 0.29	6 ± 0.46	0.025	0.04	3.2 ± 0.31	4.10E-04	0.0013
**Ethyl acetate**	9.6 ± 0.47	9.2 ± 0.57	0.6	0.65	7.6 ± 0.66	0.013	0.026
**Geosmin**	4.3 ± 0.23	5 ± 0.36	0.053	0.072	2.5 ± 0.21	7.80E-07	1.20E-05
**Hexanal**	7.5 ± 0.35	7.8 ± 0.47	0.6	0.65	5.2 ± 0.44	1.00E-04	4.10E-04
**Isoamyl acetate**	7.1 ± 0.41	7.1 ± 0.42	0.92	0.92	5.3 ± 0.59	0.013	0.026
**(Z)-3-Hexenyl acetate**	4.4 ± 0.23	5.4 ± 0.36	0.015	0.027	2.8 ± 0.22	5.90E-06	3.20E-05

Table S7. 4-way ANOVA of mean response amplitudes for mixed odours and components

	Sum Sq.	*df*	Mean Sq.	*F*	*p*
**Species**	0.0012	1	0.0012	2.8E-4	0.99
**Side**	12	1	12	2.8	0.094
**Odour**	1800	7	260	60	6.5E-86
**Glomerulus**	9400	32	290	0	1
**Species:side**	0.13	1	0.13	0.03	0.86
**Species:odour**	330	7	47	11	1.3E-13
**Species:glomerulus**	860	32	27	6.1	2.3E-25
**Side:odour**	9.6	7	1.4	0.31	0.95
**Side:glomerulus**	230	32	7.1	1.6	0.014
**Odour:glomerulus**	3300	224	15	3.3	2.4E-55
**Error**	57000	13155			
**Total**	75000	13499			

Table S8. Multiple comparison analysis of the species dependence for individual glomerular responses averaged over the mixed odours and their components

Glomerulus	-95% CI	Mean	+95% CI	*P*	P_{FDR}
**D**	-0.47	-0.012	0.44	0.96	0.96
**DA1**	-0.33	0.13	0.59	0.58	0.77
**DA2**	-0.77	-0.32	0.14	0.17	0.48
**DA3**	-0.33	0.12	0.57	0.61	0.77
**DA4l**	-0.47	-0.013	0.44	0.95	0.96
**DA4m**	-0.65	-0.2	0.25	0.38	0.74
**DC1**	-0.26	0.14	0.53	0.5	0.74
**DC2**	-0.36	0.023	0.4	0.91	0.96
**DC3**	-0.3	0.14	0.57	0.54	0.74
**DL1**	-0.53	-0.092	0.34	0.68	0.83
**DL3**	-0.75	-0.21	0.33	0.45	0.74
**DL4**	-0.35	0.23	0.8	0.44	0.74
**DL5**	0.97	1.4	1.8	1.2E-11	2E-10
**DM1**	-0.24	0.14	0.53	0.46	0.74
**DM2**	1.2	1.6	2	2.9E-17	9.5E-16
**DM3**	-0.85	-0.46	-0.066	0.022	0.1
**DM4**	-1.1	-0.71	-0.33	2.5E-4	0.0019
**DM5**	-1.1	-0.74	-0.35	1.6E-4	0.0018
**DM6**	-1.1	-0.73	-0.33	2.9E-4	0.0019
**VA1d**	-0.59	-0.086	0.41	0.74	0.87
**VA1v**	-0.83	-0.33	0.17	0.19	0.49
**VA2**	-0.37	0.023	0.42	0.91	0.96
**VA5**	-0.16	0.25	0.66	0.24	0.52
**VA6**	-0.76	-0.36	0.032	0.072	0.26
**VA7**	-0.28	0.13	0.53	0.54	0.74

**VC1**	-0.16	0.24	0.64	0.23	0.52
**VC2**	-0.52	-0.13	0.26	0.51	0.74
**VC4**	-0.39	-0.01	0.37	0.96	0.96
**VM2**	-0.074	0.31	0.7	0.11	0.37
**VM5d**	-0.12	0.26	0.64	0.18	0.48
**VM5v**	-0.92	-0.54	-0.16	0.0051	0.028
**VM7d**	-0.17	0.2	0.58	0.29	0.6
**VM7v**	-0.76	-0.38	-0.0042	0.047	0.2

Table S9. Multiple comparison analysis of the species dependence for individual mixed odours and components averaged over all glomeruli

**Odour**	**-95% CI**	**Mean**	**+95% CI**	***P***	**P_{FDR}**
**Acetic acid**	-0.34	-0.15	0.037	0.12	0.23
**Ethyl acetate**	-0.22	-0.036	0.15	0.71	0.81
**Ethyl acetate - Acetic acid**	-0.18	0.0096	0.2	0.92	0.92
**Ethyl acetate - Geosmin**	0.36	0.64	0.93	9.7E-06	3.9E-05
**Geosmin**	0.0072	0.2	0.38	0.042	0.11
**Isoamyl acetate**	-0.27	-0.08	0.11	0.41	0.65
**Isoamyl acetate - Acetic acid**	-0.87	-0.68	-0.49	1.4E-12	1.1E-11
**Isoamyl acetate - Geosmin**	-0.19	0.098	0.38	0.5	0.67

Table S10. Multiple comparison analysis of Euclidean distances between species vs. within species for odour mixtures and their components

	Between species	*D. melanogaster*			*D. suzukii*		
	**Mean ± SEM**	**Mean ± SEM**	***P***	P_{FDR}	**Mean ± SEM**	***P***	P_{FDR}
**Acetic acid**	4.9 ± 0.29	6 ± 0.46	0.025	0.04	3.2 ± 0.31	4.10E-04	3.30E-03
**Ethyl acetate**	9.6 ± 0.47	9.2 ± 0.57	0.6	0.64	7.6 ± 0.66	0.013	0.023
**Ethyl acetate - Acetic acid**	8.7 ± 0.48	8.2 ± 0.51	0.52	0.6	7.1 ± 0.71	5.70E-02	0.076
**Ethyl acetate - Geosmin**	2 ± 0.26	3.6 ± 0.52	0.0025	0.0099	0.82 ± 0.19	0.0016	0.0084
**Geosmin**	4.3 ± 0.23	5 ± 0.36	0.053	0.076	2.5 ± 0.21	7.80E-07	1.20E-05
**Isoamyl acetate**	7.1 ± 0.41	7.1 ± 0.42	0.92	0.92	5.3 ± 0.59	1.30E-02	2.30E-02
**Isoamyl acetate - Acetic acid**	8.7 ± 0.46	6.8 ± 0.39	0.0065	0.015	7.4 ± 0.69	0.1	0.13
**Isoamyl acetate - Geosmin**	1.4 ± 0.18	2.5 ± 0.38	0.0033	0.01	0.62 ± 0.15	5.60E-03	1.50E-02

Table S11. 2-way ANOVA of the odour- and species-dependence for 3 behavioural experiments each with one different reference odour (bait1)

Bait1 = Acetic acid

	Sum Sq.	*df*	Mean Sq.	*F*	*p*
**bait2**	0.83	2	0.42	36	6.3E-11
**species**	0.000074	1	0.000074	0.0065	0.94
**bait2:species**	0.0064	2	0.0032	0.28	0.76
**Error**	0.67	58	0.012		
**Total**	1.5	63			

Bait1 = Ethyl acetate

	Sum Sq.	*df*	Mean Sq.	*F*	*p*
**bait2**	3.7	4	0.91	77	4.1E-38
**species**	0.01	1	0.01	0.86	0.35
**bait2:species**	0.2	4	0.051	4.3	0.0025
**Error**	2.2	182	0.012		
**Total**	6	191			

Bait1 = Isoamyl acetate

	Sum Sq.	*df*	Mean Sq.	*F*	*p*
**bait2**	2.3	4	0.58	30	2.8E-19
**species**	0.0029	1	0.0029	0.15	0.7
**bait2:species**	0.073	4	0.018	0.94	0.44
**Error**	3.5	182	0.019		
**Total**	5.9	191			

Table S12. Multiple comparisons of odour- and species-dependence for 3 behavioural experiments each with a different reference odour (Bait1)

D. suzukii vs D. melanogaster	**-95% CI**	**mean**	**+95% CI**	***P***	P_{FDR}
**Bait1 = Acetic acid**					
Acetic acid	-0.083	-0.0068	0.069	0.86	0.91
Empty	-0.074	0.033	0.14	0.54	0.78
Paraffin oil	-0.13	-0.02	0.088	0.72	0.85
**Bait1 = Ethyl acetate**					
Empty	-0.098	-0.036	0.026	0.26	0.48
Ethyl acetate	-0.00032	0.053	0.11	0.051	0.33
Paraffin oil	-0.15	-0.088	-0.026	0.0059	0.076
Acetic acid	-0.021	0.087	0.19	0.11	0.48
Ethyl acetate + Acetic acid	-0.038	0.07	0.18	0.2	0.48
**Bait1 = Isoamyl acetate**					
Acetic acid	-0.054	0.083	0.22	0.24	0.48
Empty	-0.075	0.0044	0.083	0.91	0.91
Isoamyl acetate	-0.11	-0.041	0.028	0.24	0.48
Paraffin oil	-0.045	0.034	0.11	0.4	0.65
Isoamyl acetate + Acetic acid	-0.17	-0.034	0.1	0.62	0.81

Table S13 Within-species multiple comparisons of behavioural odour preferences. Comparisons with controls are not shown as odours were always significantly preferred over paraffin oil, which in turn was significantly preferred over the empty vial.

Bait1 = Ethyl acetate

**Bait2**	**Bait2**	**-95% CI**	**mean**	**+95% CI**	***p***	***p_{FDR}***
D. melanogaster						
Ethyl acetate	Acetic acid	-0.076	0.0092	0.094	0.83	0.83
Ethyl acetate	Ethyl acetate + Acetic acid	-0.21	-0.12	-0.036	0.0056	0.0081
Acetic acid	Ethyl acetate + Acetic acid	-0.24	-0.13	-0.022	0.018	0.02
D. suzukii						
Ethyl acetate	Acetic acid	-0.043	0.042	0.13	0.33	0.33
Ethyl acetate	Ethyl acetate + Acetic acid	-0.19	-0.1	-0.019	0.016	0.02
Acetic acid	Ethyl acetate + Acetic acid	-0.25	-0.15	-0.039	0.0077	0.011

Bait1 = Isoamyl acetate

**Bait2**	**Bait2**	**-95% CI**	**mean**	**+95% CI**	***p***	***p_{FDR}***
D. melanogaster						
Acetic acid	Isoamyl acetate	0.014	0.12	0.23	0.027	0.034
Acetic acid	Isoamyl acetate + Acetic acid	-0.17	-0.038	0.099	0.59	0.59
Isoamyl acetate	Isoamyl acetate + Acetic acid	-0.27	-0.16	-0.052	0.004	0.0067
D. suzukii						
Acetic acid	Isoamyl acetate	0.014	0.25	0.36	1.4E-5	3.4E-5
Acetic acid	Isoamyl acetate + Acetic acid	-0.058	0.079	0.22	0.26	0.27
Isoamyl acetate	Isoamyl acetate + Acetic acid	-0.28	-0.17	-0.059	0.0028	0.004
